# HEAVEN criteria to predict difficult in-hospital rapid sequence intubation: a prospective single-centre observational study

**DOI:** 10.1097/EJA.0000000000002382

**Published:** 2026-03-06

**Authors:** Alexander Fuchs, Charlotte Backhauss, Lea Franzmeier, Claudio Bonesana, Daniele Malpetti, Lea Ruscher, Markus Huber, Thomas Riva, Laura Azzimonti, Robert Greif

**Affiliations:** From the Department of Anaesthesiology and Pain Medicine, Inselspital, Bern University Hospital, University of Bern, Bern (AF, CB, LF, LR, MH, TR), Dalle Molle Institute for Artificial Intelligence (IDSIA), SUPSI, Lugano (CB, DM, LA), and Medical Faculty, University of Bern, Bern, Switzerland (RG)

Editor,

Airway assessment is recommended to minimise unanticipated airway management difficulties.^1,2^ Patients with life-threatening conditions often present with hypoxaemia and haemodynamic instability.^3^ Furthermore, these patients are frequently not fasted or have delayed gastric emptying, heightening the risk of pulmonary aspiration of gastric contents during tracheal intubation.^2^ Rapid sequence intubation (RSI) aims to minimise this risk. Assessment tools used to predict airway management difficulty have low predictive power and often require patient co-operation.^4^ Most assessment tools do not consider physiological factors. The HEAVEN criteria (Hypoxaemia, Extremes of size, Anatomical abnormalities, Vomit/blood/fluid, Exsanguination, Neck mobility issues) were developed and validated to predict difficulties during out-of-hospital tracheal intubation without patient co-operation.^5^ Physiological challenges, including anaemia and oxygen dependency, and anatomical challenges, including limited neck mobility and altered airway anatomy, can be present in patients undergoing in-hospital RSI, especially for an emergency indication. The predictive performance of HEAVEN on difficult intubation in the in-hospital environment has not been systematically evaluated. We examined whether the HEAVEN criteria could serve as a reliable bedside test for predicting difficult intubation in the in-hospital setting.

This single-centre prospective observational study was conducted at a tertiary university hospital in Switzerland with approval of the responsible Cantonal Ethics Committee of Bern (BASEC 2020-02458) and prospective registration (NCT04764799). All adult and paediatric patients undergoing RSI between 01 December 2021 and 30 July 2023 were included, with only patients in cardiac arrest or missing the primary outcome data excluded. Indications for RSI included fasting of solid food content less than 6 h, gastrointestinal pathology, pregnancy at least 12 weeks’ gestation, nausea or vomiting, or recent trauma. The patients were grouped according to the urgency of airway management required. Patients assigned to the *emergency group* were those who presented with a potentially life-threatening situation that required immediate RSI. All patients received a neuromuscular blocking agent at induction. The airway management device was either a video laryngoscope with standard or hyperangulated blade (C-MAC, Karl Storz, Tuttlingen, Germany) or a direct laryngoscope (HEINE Optotechnik, Germany). Baseline characteristics, HEAVEN criteria,^5^ number of intubation attempts and difficulty of intubation ^6^ were recorded in the electronic anaesthesia record and transferred to a research database (REDCap, Vanderbilt University, Nashville, Tennessee, USA). The HEAVEN criteria were recorded as previously defined: Hypoxaemia, Extremes of size (BMI >35 or age <8y), Anatomical abnormalities, Vomit/blood/fluid, Exsanguination (acute blood loss or chronic anaemia haemoglobin <100 g l^−1^) and neck mobility issues.^5^

The associations of the HEAVEN criteria with the primary outcomes (a) first-attempt success and (b) difficulty of intubation were assessed using two separate logistic regression models: The individual model included the six HEAVEN criteria as covariates; the cumulative model used the total number of HEAVEN criteria as an ordinal covariate. For each primary outcome, separate models were fitted for the emergency and nonemergency cohorts, and, within each cohort, for the intubation device (video laryngoscope or direct laryngoscope). Model performance was evaluated using the area under the receiver operating characteristic curve (AUROC). For each primary outcome, we calculated sensitivity and specificity and, based on the prevalence, the positive predictive value (PPV) and the negative predictive value (NPV). We performed a complete case analysis of all patients for whom first-attempt success and difficult RSI were recorded. All analyses were performed with R version 4.0.2.

In total, we included 3517 patients, of which 16.5% had an emergency indication. The patient characteristics and outcomes are provided in Supplemental Table S1. First-attempt intubation success was lower (93.2 vs. 94.9%), and difficult RSI was more frequent (9.6 vs. 5.1%) in the emergency cohort than in the nonemergency cohort. Video laryngoscope was associated with a higher first-attempt intubation success rate compared with direct laryngoscope (94.3 vs. 92.2%, *P* = 0.008).

Figure 1 illustrates the relationship between HEAVEN criteria and the primary outcomes in emergency patients. With higher numbers of cumulative HEAVEN criteria, the risk probability of first-attempt success decreased while the difficulty of intubation increased. In the model using individual criteria, Anatomic challenges and Vomit/blood/fluid were associated with a lower adjusted risk for first-attempt success. Anatomic challenges, Vomit/blood/fluid and Exsanguination were associated with an adjusted risk increase of difficulty of intubation.

**Fig. 1:**
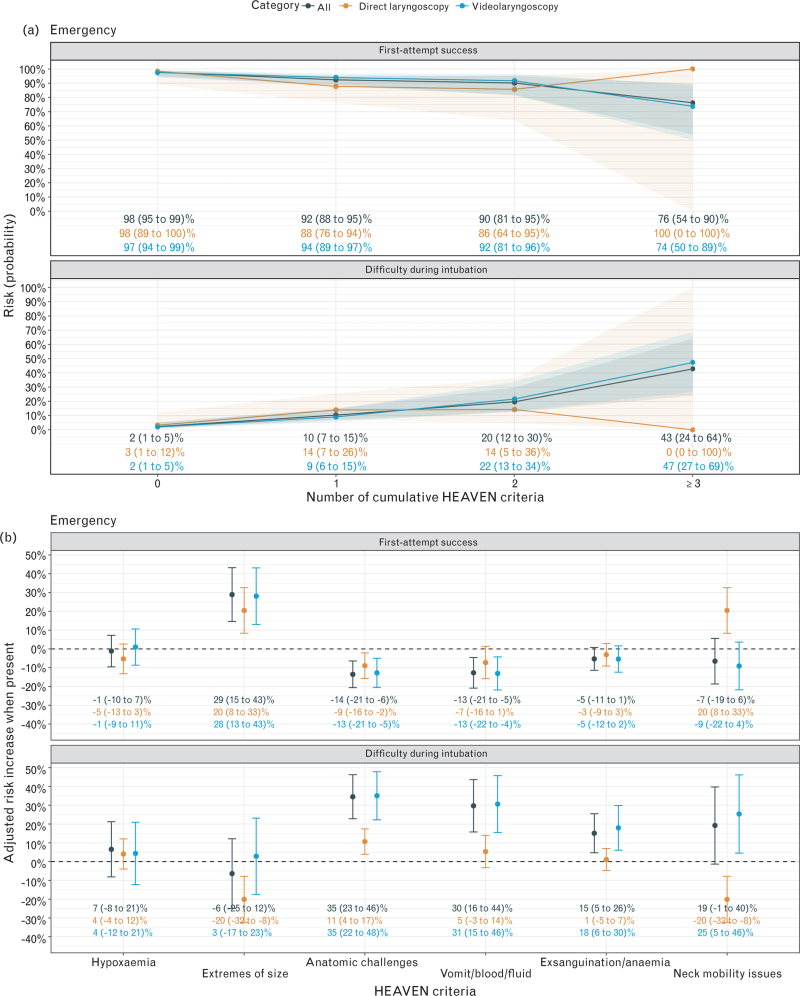
(a) Predicted risk (probability) of first-attempt success and difficulty during rapid sequence intubation (RSI), displayed for the overall cohort and stratified by direct and video laryngoscopy across increasing numbers of HEAVEN criteria. (b) Adjusted risk increase for first-attempt success and difficulty during RSI associated with each individual HEAVEN criterion, stratified by intubation device. Data represent mean estimates with 95% confidence intervals.

In the emergency cohort, for first-attempt success, the area under the receiver operating characteristic (AUROC) of the individual HEAVEN criteria was 0.77 (95% CI 0.69 to 0.85). The individual HEAVEN criteria had a sensitivity of 99.8% (95% CI 99.0 to 100.0) and a specificity of 11.1% (95% CI 3.1 to 26.1). The PPV was 94.5% (95% CI 99.2 to 96.2) and the NPV 80.0% (95% CI 28.4 to 99.5). For difficult RSI, the AUROC for the individual HEAVEN criteria was 0.79 (95% CI 0.73 to 0.86). The individual HEAVEN criteria had a sensitivity of 0.0% (95% CI 0.0 to 6.6) and a specificity of 100.0% (95% CI 99.3 to 100.0). The PPV was ‘Not a Number’% (95% CI 0.0 to 100.0) and the NPV was 90.7% (95% CI 88.0 to 92.9). The HEAVEN criteria showed lower performance metrics in the nonemergency cohort and with the cumulative model (Digital Supplement).

The summary sensitivity and specificity of commonly reported bedside predicting tools for difficult intubation with the modified Mallampati score were 0.51 (95% CI 0.40 to 0.61) and 0.87 (95% CI 0.82 to 0.91), and with the Mouth opening, 0.27 (95% CI 0.16 to 0.41) and 0.93 (95% CI 0.87 to 0.96).^4^ Given that these tools require patient co-operation and are often not feasible in emergency scenarios, the HEAVEN criteria provide a practical alternative for assessing the airway in unconscious or unco-operative patients (e.g. dementia). However, the HEAVEN criteria showed low predictive performance for either primary outcome. Given the low number of patients presenting with three or more criteria, the clinical utility of the HEAVEN criteria for in-hospital emergency RSI may be limited.

For paediatric tracheal intubation, the use of a video laryngoscope ^7^ and apnoeic oxygenation ^8^ can increase first-attempt success and are standard practice at the study's institution. Similar evidence exists for tracheal intubation in adult patients living with obesity.^9,10^

In contrast to the out-of-hospital studies investigating the HEAVEN criteria,^5^ we observed significantly higher first-attempt success rates. Thus, we tested the HEAVEN criteria on a low-incidence event in our setting, which markedly reduced the predictive performance. Although the presence of HEAVEN criteria had limited predictive value, their absence reliably predicted a high likelihood of first-attempt intubation success and ease of intubation.

Our study had several limitations. The single-centre design may not be generalisable to other healthcare settings, particularly with respect to the prevalence of first-attempt success and the incidence of intubation difficulties. Furthermore, confounders, such as the use of video laryngoscopy, might have improved glottic visualisation and ease of intubation. Finally, first-attempt tracheal intubation success and difficulties during RSI depend on the airway operator's experience, which was not standardised in our setting.

In this study of patients undergoing in-hospital RSI with emergency and nonemergency indications, individual HEAVEN criteria showed stronger predictive value than the cumulative model. For first-attempt success, these were Anatomic challenges, Vomit/blood/fluid, and for difficult RSI, Anatomic challenges, Vomit/blood/fluid and Exsanguination. The HEAVEN criteria were not a reliable standalone assessment tool for first-attempt success or difficult RSI in either cohort. Our data suggest that the absence of HEAVEN criteria was associated with higher first-attempt intubation success and greater ease during RSI in emergency and nonemergency patients.

## Supplementary Material

Supplemental Digital Content
