# Supplementary material for: HEAVEN criteria to predict difficult in-hospital rapid sequence intubation: a prospective single-centre observational study
Source: Eur J Anaesthesiol. 2026 Mar 6;43(6):550–3. doi: 10.1097/EJA.0000000000002382 (PMC13152049; doi:10.1097/EJA.0000000000002382)
Supplement: Supplemental Digital Content [file ejanet-43-550-s001.docx]

**Supplemental Material**

**Extended Methods Section**

*Variables*

We recorded patients' baseline characteristics (age, sex, height, weight, American Society of Anesthesiologists (ASA) physical status) and airway assessment (modified Mallampati). For each RSI, we recorded the number of intubation attempts and the difficulty of intubation on a three-point scale (easy, difficult, and not possible)^1^. The percentage of glottic opening (POGO) score^2^ on a five-point scale (0%, 1%-25%, 26-50%, 51-75%, 76%-100%), and the use of external laryngeal manoeuvres to improve glottic visualisation were captured. The operator’s level of experience (consultant, resident, nurse), including the number of practice years, and any changes in the airway operator during the RSI were recorded.

The airway management device was either a video laryngoscope with standard or hyperangulated blade (C-MAC, Karl Storz, Tuttlingen, Germany), or a direct laryngoscope (Heine, HEINE Optotechnik GmbH & Co. KG, Germany).

The HEAVEN criteria were recorded as previously defined^3^:

**Hypoxaemia**: SpO2 ≤90% at initial laryngoscopy or patients with underlying pulmonary conditions requiring pre-induction supplemental oxygen (i.e. nasal oxygen or non-invasive ventilation)

**Extremes of Size**: Paediatric patients (≤8 years of age) or patients with a BMI >35 or clinical obesity, as determined by the operator when the BMI was unknown.

**Anatomic Challenges**: Any structural abnormality anticipated to limit laryngoscopic view, such as trauma to airway structures, limited oral aperture, large tongue, short neck, mass lesions, swelling, foreign bodies, or external structures obstructing visualisation. Furthermore, temporomandibular joint dysfunction, previous head and neck surgeries, or conditions that alter normal airway anatomy, such as thyroid masses or congenital abnormalities, were also considered.

**Vomit/ Blood/ Fluid**: Clinically significant fluid noted in the pharynx or hypopharynx before laryngoscopy. Presence of blood or fluid, particularly in high-risk procedures with lower urgency (e.g. upper gastrointestinal bleeding)

**Exsanguination**: Anaemia, either chronic (based on past medical history or laboratory Haemoglobin values <100 g l^-1^) or acute (based on chief complaint, mechanism of injury, or examination findings).

**Neck Mobility Issues**: Patients with limited and restricted cervical range of motion, including cervical spine injury, arthritis, or prior cervical spine surgery.

*Procedure and measure*

The patients were categorised according to the urgency of the procedure. Patients in the *emergency group* presented with a potentially life-threatening situation requiring immediate RSI without any time delay, not fastened for 6 hours. These patients were intubated under high time pressure for the team. Patients in the *non-emergency group* were either non-scheduled interventions with lower urgency or elective cases. The airway operator recorded all variables in the anaesthesia electronic health record (AIS, Copra System, Berlin, Germany). The data were automatically extracted daily from the anaesthesia information system and entered into a dedicated departmental research database (REDCap, Vanderbilt University, Nashville, Tennessee, USA). After data transfer, all entries were manually checked in the database, and in case of missing data, the documented airway operator was directly interviewed by the research team.

*Primary outcomes*

The primary outcomes were the predictive capacity of the HEAVEN criteria^3^ for the first-attempt tracheal intubation success and difficulty during intubation. An intubation attempt started whenever the laryngoscope blade passed the lips and ended with the measurement of the first end-tidal CO_2_. The difficulty during intubation was recorded on a three-point scale (easy, difficult, and not possible) using the definitions of the Fremantle Score^1^ and the Video Classification of Intubation (VCI) score^4^. *Easy* was defined as a smooth intubation on the first attempt without changing the device (i.e. changing the laryngoscope blade or unplanned adjuncts such as a stylet or bougie) or patient positioning. *Difficult* was defined as a change of device, unplanned use of adjuncts (i.e. stylet or bougie) or repositioning of the patient. *Not possible* was defined as tracheal intubation that was impossible and required an alternative airway strategy (i.e., supraglottic airway or emergency front-of-neck access).

*Statistical analysis*

For the sample size calculation, we followed the framework of Peduzzi et al.^5^ to calculate the minimum number of cases for reliable logistic regression: N = 10*k/p, where k denotes the number of covariates and p the smallest of the successful or unsuccessful cases. For the six HEAVEN criteria (k=6) and p=7%-10% (*first-attempt success:* 7%, *difficulty during intubation*: 10%)^6^, we derived a sample size ranging from 600 to 858 patients for each urgency category (emergency vs non-emergency). We increased the total sample size to 2,800, anticipating a higher number of non-emergency RSIs.

Categorical variables are presented as counts and frequencies. Continuous variables are summarised with mean and standard deviation or median and IQR, depending on distribution.

The associations of the HEAVEN criteria with the primary outcomes, *first-attempt success* (yes=1 or no=0), and *difficulty of intubation (*easy=0 or difficult=1) were assessed using two logistic regression models. The first model included the six HEAVEN criteria as covariates. The second model used the total number of HEAVEN criteria as an ordinal covariate. Separate models were fitted for the emergency and non-emergency cohorts. **M**odel performance was evaluated using the area under the receiver operating characteristic curve (AUROC). For the metrics of each primary outcome, we calculated the sensitivity and the specificity. Based on the prevalence of each primary outcome, we calculated the positive predictive value (PPV) and the negative predictive value (NPV).

We performed a complete case analysis of all patients in which the *first-attempt success* and the *difficulty during intubation* were recorded.

All analyses were performed with R version 4.0.2.^7^

**Results**

We screened 4994 patients undergoing RSI over the 20-month observation period, and 3517 patients (16.5%, n=580 emergency and 83.5%, n=2927 non-emergency) were included **(Supplemental Figure S1)**. The median age was 63 [39 to 77] years in the emergency cohort (44.5% female) and 55 [34 to 70] years in the non-emergency cohort (50.2% female) **(Supplemental Table S1)**.

**Supplemental Figure S2** displays the distribution of cumulative HEAVEN criteria (A, C) and the number and percentage of each criterion for emergency and non-emergency patients (B, D), both according to the intubation device (video or direct laryngoscope).

*Non-emergency cohort*

Among the non-emergency cohort (**Supplemental Table S2**), for *first-attempt success,* the area under the receiver operating characteristic (AUROC) of the individual HEAVEN criteria was 0.67 (95% CI: 0.63-0.72) for both intubation devices and was slightly higher for the video laryngoscope **(Supplemental Figure S3 and Supplemental Table S4)**. The individual HEAVEN criteria had a sensitivity of 100.0%(95%CI: 99.8 to 100.0) and a specificity of 1.4%(95%CI: 0.2 to 5.1). The corresponding PPV was 95.3%(95%CI: 94.5 to 96.0) and the NPV 66.7%(95%-CI: 9.4 to 99.2).

Among the non-emergency cohort, for *difficult RSI,* the AUROC of the individual HEAVEN criteria was 0.76(95%CI: 0.72 to 0.80). The individual HEAVEN criteria had a sensitivity of 1.3%(95%CI: 0.2 to 4.8) and a specificity of 100.0%(95%CI: 99.8 to 100.0). The corresponding PPV was 66.7%(95%CI: 9.4 to 99.2) and the NPV 95.0%(95%-CI: 94.1 to 95.8).

Compared with the model using individual HEAVEN criteria, the cumulative model had lower AUCs for both primary outcomes in both emergency and non-emergency patients (**Supplemental Table S4, Supplemental Figure S4)**.

The baseline risk of *first-attempt success* did not differ between the emergency and non-emergency cohorts (**Supplemental Figure 2**). In contrast, the probability of *difficult RSI*, excluding predictive variables such as the HEAVEN criteria, was significantly higher in the emergency cohort than in the non-emergency cohort.

**References**

1. O'Loughlin EJ, Swann AD, English JD, Ramadas R. Accuracy, intra- and inter-rater reliability of three scoring systems for the glottic view at videolaryngoscopy. *Anaesthesia* 2017; **72**:835-839.

2. Levitan RM, Ochroch EA, Kush S, Shofer FS, Hollander JE. Assessment of airway visualization: validation of the percentage of glottic opening (POGO) scale. *Acad Emerg Med* 1998; **5**:919-923.

3. Davis DP, Olvera DJ. HEAVEN Criteria: Derivation of a New Difficult Airway Prediction Tool. *Air Med J* 2017; **36**:195-197.

4. Chaggar RS, Shah SV, Berry M, Saini R, Soni S, Vaughan D. The Video Classification of Intubation (VCI) score: a new description tool for tracheal intubation using videolaryngoscopy: A pilot study. *Eur J Anaesthesiol* 2021; **38**:324-326.

5. Peduzzi P, Concato J, Kemper E, Holford TR, Feinstein AR. A simulation study of the number of events per variable in logistic regression analysis. *J Clin Epidemiol* 1996; **49**:1373-1379.

6. Schweizer T., Hugger S., Kaluza P., Theiler L., Greif R. HEAVEN criteria: prediction of difficult airway during in-hospital rapid sequence intubations. European Journal of Anaesthesiology [online serial] June 2020; Volume 37: e-Supplement 58. <https://journals.lww.com/ejanaesthesiology/Documents/ESA_Abstracts_2020.pdf>.

7. R Core Team, R: A language and environment for statistical computing. *R Foundation for Statistical Computing, Vienna, Austria* 2021.

**Supplemental Table and Figure Legend**

**Supplemental Table S1.** Patients’ baseline characteristics undergoing emergency or non-emergency in-hospital rapid sequence intubation for direct laryngoscopy (DL) and video laryngoscopy (VL). Values are number (proportion) or median [Q1; Q3]. P-values refer to the comparison between DL and VL.

**Supplemental Table S2.** Primary outcomes and airway procedural data of patients undergoing emergency or non-emergency in-hospital rapid sequence intubation with direct laryngoscopy (DL) or video laryngoscopy (VL). Values are number (proportion) or median [Q1; Q3]. P-values refer to the group comparison between emergency and non-emergency patients.

**Supplemental Table S3.** Area under the receiver operating characteristic (AUROC) for the primary outcomes, *first-attempt success* and *difficulty of intubation,* for the individual and cumulative HEAVEN criteria model for the emergency and non-emergency cohort undergoing rapid sequence intubation with direct or video laryngoscopy

**Supplemental Table S4.** Sensitivity, specificity, positive prediction value (PPV), and negative prediction value (NPV) of the HEAVEN criteria for the primary outcomes, *first-attempt success* and *difficulty of intubation,* for the individual and cumulative HEAVEN criteria model for the emergency and non-emergency cohort undergoing rapid sequence intubation with direct or video laryngoscopy

**Supplemental Table S5.** Airway operator characteristics for paediatric patients and adult patients in the emergency and non-emergency cohorts

**Supplemental Figure S1.** Study Flow Chart

**Supplemental Figure S2.** The distribution of cumulative HEAVEN criteria (A, C) and the number and percentage of each criterion for emergency and non-emergency patients (B, D), both according to the intubation device (video or direct laryngoscope)

**Supplemental Figure S3. A**: Risk (probability) of first-attempt success and difficulties during rapid sequence intubation in emergency patients for the overall cohort and stratified by direct and video laryngoscopy across increasing numbers of cumulative HEAVEN criteria. Increasing numbers of HEAVEN criteria are associated with lower first pass success and higher risk of difficult airway. **B**: Adjusted risk increase associated with each individual HEAVEN criterion for first pass success and difficult airway in emergency patients, stratified by intubation device. Panel A is based on a prediction model including the cumulative number of HEAVEN criteria (cumulative model), and Panel B on a model including individual HEAVEN criteria (individual model). Data are presented as mean risk estimates with 95% confidence intervals; corresponding numerical values are shown below each panel. Wide 95% confidence intervals for direct laryngoscopy with more than three criteria indicate infrequent use of this device in such cases and limit interpretation.

**Supplemental Figure S4.** Area under the receiver operating characteristic (AUROC) for the primary outcomes (A) *difficulty of intubation*, and (B) *first-attempt success* for the individual and cumulative HEAVEN criteria model.

**Supplemental Table S1.** Patients’ baseline characteristics undergoing emergency or non-emergency in-hospital rapid sequence intubation for direct laryngoscopy (DL) and video laryngoscopy (VL). Values are number (percent) or median [IQR]. *P* values refer to the comparison between DL and VL.

|  | **All** | | **Emergency** | | | |  | **Non-Emergency** | | | |  |
| --- | --- | --- | --- | --- | --- | --- | --- | --- | --- | --- | --- | --- |
|  |  | **N** | **All** | **DL** | **VL** | **N** | ***P*** | **All** | **DL** | **VL** | **N** | ***P*** |
|  | ***N=3517*** |  | ***N=580*** | ***N=141*** | ***N=439*** |  |  | ***N=2937*** | ***N=822*** | ***N=2115*** |  |  |
| **Age**, yr | 56.0 [35.0 to 71.0] | 3517 | 63.0 [39.0 to 78.0] | 63.0 [36.0 to 78.] | 64.0 [39.0 to 77.0] | 580 | 0.655 | 55.0 [34.0 to 70.0] | 49.0 [22.2 to 68.0] | 56.0 [37.0 to 70.0] | 2937 | <0.001 |
| **Sex,** female | 1740 (49.5) | 3517 | 258 (44.5) | 55 (39.0) | 203 (46.2) | 580 | 0.145 | 1482 (50.5) | 410 (49.9) | 1072 (50.7) | 2937 | 0.711 |
| **Height,** cm | 170 [162 to 176] | 3184 | 170 [165 to 177] | 172 [165 to 178] | 170 [165 to 176] | 409 | 0.650 | 169 [162 to 176] | 168 [160to 175] | 170 [163 to 176] | 2775 | <0.001 |
| **Weight**, kg | 74.0 [61.0 to 87.5] | 3495 | 75.0 [65.0 to 85.0] | 75.0 [64.2 to 85.0] | 75.0 [65.0 to 85.0] | 564 | 0.574 | 73.0 [60.0 to 88.0] | 69.0 [53.0 to 84.0] | 75.0 [63.0 to 89.0] | 2931 | <0.001 |
| **BMI**, kg.m^-2^ | 25.6 [22.1 to 30.0] | 3181 | 25.9 [23.1 to 28.7] | 25.9 [23.4 to 28.9] | 25.9 [23.1 to 28.6] | 409 | 0.810 | 25.5 [22.0 to 30.1] | 24.6 [20.9 to 29.1] | 25.8 [22.4 to 30.4] | 2772 | <0.001 |
| **ASA physical status** |  | 3517 |  |  |  | 580 | 0.187 |  |  |  | 2937 | <0.001 |
| 1 | 312 (8.87) |  | 25 (4.31) | 9 (6.38) | 16 (3.64) |  |  | 287 (9.77) | 122 (14.8%) | 165 (7.80%) |  |  |
| 2 | 1115 (31.7) |  | 105 (18.1) | 23 (16.3) | 82 (18.7) |  |  | 1010 (34.4) | 295 (35.9%) | 715 (33.8%) |  |  |
| 3 | 1371 (39.0) |  | 130 (22.4) | 36 (25.5) | 94 (21.4) |  |  | 1241 (42.3) | 303 (36.9%) | 938 (44.3%) |  |  |
| 4 | 654 (18.6) |  | 262 (45.2) | 55 (39.0) | 207 (47.2) |  |  | 392 (13.3) | 100 (12.2%) | 292 (13.8%) |  |  |
| 5 | 65 (1.85) |  | 58 (10.0) | 18 (12.8) | 40 (9.11) |  |  | 7 (0.24) | 2 (0.24%) | 5 (0.24%) |  |  |
| **Modified Mallampati score** |  | 3517 |  |  |  | 580 | 0.264 |  |  |  | 2937 | 0.020 |
| I | 1007 (28.6) |  | 77 (13.3) | 16 (11.3) | 61 (13.9) |  |  | 930 (31.7) | 270 (32.8) | 660 (31.2) |  |  |
| II | 1318 (37.5) |  | 124 (21.4) | 23 (16.3) | 101 (23.0) |  |  | 1194 (40.7) | 325 (39.5) | 869 (41.1) |  |  |
| III | 396 (11.3) |  | 35 (6.0) | 10 (7.1) | 25 (5.7) |  |  | 361 (12.3) | 86 (10.5) | 275 (13.0) |  |  |
| IV | 72 (2.05) |  | 9 (1.6) | 1 (0.71) | 8 (1.8) |  |  | 63 (2.15) | 12 (1.5) | 51 (2.4) |  |  |
| Not assessable | 724 (20.6) |  | 335 (57.8) | 91 (64.5) | 244 (55.6) |  |  | 389 (13.2) | 129 (15.7) | 260 (12.3) |  |  |
| **HEAVEN criteria** |  | 3517 |  |  |  | 580 |  |  |  |  | 2937 |  |
| Hypoxaemia | 134 (3.8) |  | 62 (10.7) | 13 (9.2) | 49 (11.2) |  | 0.622 | 72 (2.5) | 17 (2.1) | 55 (2.6) |  | 0.481 |
| Extremes of size | 519 (14.8) |  | 54 (9.31) | 23 (16.3) | 31 (7.1) |  | 0.002 | 465 (15.8) | 143 (17.4) | 322 (15.2) |  | 0.164 |
| Anatomic abnormalities | 377 (10.7) |  | 80 (13.8) | 16 (11.3) | 64 (14.6) |  | 0.408 | 297 (10.1) | 64 (7.8) | 233 (11.0) |  | 0.011 |
| Vomit/blood/fluid | 111 (3.2) |  | 49 (8.5) | 10 (7.1) | 39 (8.9) |  | 0.623 | 62 (2.1) | 13 (1.6) | 49 (2.3) |  | 0.271 |
| Exsanguination /anaemia | 936 (26.6) |  | 184 (31.7) | 40 (28.4) | 144 (32.8) |  | 0.379 | 752 (25.6) | 181 (22.0) | 571 (27.0) |  | 0.006 |
| Neck mobility issues | 188 (5.4) |  | 21 (3.6) | 3 (2.1) | 18 (4.1) |  | 0.406 | 167 (5.7) | 24 (2.9) | 143 (6.8) |  | <0.001 |

***** Non-emergency ASA 5 patients were patients with severe, life-limiting conditions and a procedure considered critical for survival (e.g. advanced terminal illnesses with severe multi-organ failure, or ruptured aortic aneurysms stabilised after fluid resuscitation, and subsequently elective intubation 6-12 hours after hospital admission).

**Supplemental Table S2.** Primary outcomes and airway procedural data of patients undergoing emergency or non-emergency in-hospital rapid sequence intubation with direct laryngoscopy (DL) or video laryngoscopy (VL). Values are number (percent) or median [IQR]. P values refer to the group comparison between emergency and non-emergency patients.

|  |  | **Emergency** | | | | **Non-Emergency** | | | |
| --- | --- | --- | --- | --- | --- | --- | --- | --- | --- |
|  | **All** | **All** | **DL** | **VL** | ***P*** | **All** | **DL** | **VL** | ***P*** |
|  | ***N=3517*** | ***N=580*** | ***N=141*** | ***N=439*** |  | ***N=2937*** | ***N=822*** | ***N=2115*** |  |
| ***Number of intubation attempts*** | |  |  |  | 0.008 |  |  |  | 0.273 |
| 1 | 3341 (95.0) | 544 (93.8) | 130 (92.2) | 414 (94.3) |  | 2797 (95.2) | 792 (96.4) | 2005 (94.8) |  |
| 2 | 153 (4.4) | 31 (5.3) | 7 (5.0) | 24 (5.5) |  | 122 (4.2) | 28 (3.4) | 94 (4.4) |  |
| 3 | 20 (0.6) | 4 (0.7) | 4 (2.8) | 0 (0) |  | 16 (0.5) | 2 (0.2) | 14 (0.7) |  |
| 4 | 2 (0.06) | 0 (0) | 0 (0) | 0 (0) |  | 2 (0.07) | 0 (0) | 2 (0.1) |  |
| 5 | 0 (0) | 0 (0) | 0 (0) | 0 (0) |  | 0 (0) | 0 (0) | 0 (0) |  |
| >5 | 1 (0.03) | 1 (0.3) | 0 (0) | 1 (0.2) |  | 0 (0) | 0 (0) | 0 (0) |  |
| ***Intubation Difficulty*** |  |  |  |  | >0.99 |  |  |  | 0.005 |
| Easy | 3314 (94.2) | 526 (90.7) | 128 (90.8) | 398 (90.7) |  | 2788 (94.9) | 795 (96.7) | 1993 (94.2) |  |
| Difficult | 203 (5.8) | 54 (9.3) | 13 (9.2) | 41 (9.3) |  | 149 (5.1) | 27 (3.3) | 122 (5.8) |  |
| Not possible | 0 (0) | 0 (0) | 0 (0) | 0 (0) |  | 0 (0) | 0 (0) | 0 (0) |  |
| ***Device*** |  |  |  |  | <0.001 |  |  |  | 0.000 |
| Direct laryngoscope | 963 (27.4) | 141 (24.3) | 141 (100) | 0 (0) |  | 822 (28.0) | 822 (100) | 0 (0) |  |
| Video laryngoscope, standard blade | 2294 (65.2) | 387 (66.7) | 0 (0) | 387 (88.2) |  | 1907 (64.9) | 0 (0) | 1907 (90.2) |  |
| Video laryngoscope, hyperangulated blade | 260 (7.39) | 52 (9.0) | 0 (0) | 52 (11.8) |  | 208 (7.1) | 0 (0) | 208 (9.8) |  |
| ***POGO Score*** |  |  |  |  | 0.579 |  |  |  | 0.983 |
| 0% | 10 (0.3) | 4 (0.7) | 1 (0.7) | 3 (0.7) |  | 6 (0.2) | 1 (0.1) | 5 (0.2) |  |
| 1% - 25% | 48 (1.4) | 10 (1.7) | 3 (2.1) | 7 (1.6) |  | 38 (1.3) | 12 (1.5) | 26 (1.2) |  |
| 26% - 50% | 106 (3.0) | 22 (3.8) | 7 (5.0) | 15 (3.4) |  | 84 (2.9) | 23 (2.8) | 61 (2.9) |  |
| 51% - 75% | 448 (12.7) | 58 (10.0) | 11 (7.8) | 47 (10.7) |  | 390 (13.3) | 112 (13.6) | 278 (13.1) |  |
| > 75% | 2601 (74.0) | 424 (73.1) | 100 (70.9) | 324 (73.8) |  | 2177 (74.1) | 605 (73.6) | 1572 (74.3) |  |
| Missing | 304 (8.6) | 62 (10.7) | 19 (13.5) | 43 (9.8) |  | 242 (8.2) | 69 (8.4) | 173 (8.2) |  |
| *External laryngeal manoeuvre applied, yes* | 678 (19.3) | 108 (18.6) | 26 (18.4) | 82 (18.7) | >0.99 | 570 (19.4) | 174 (21.2) | 396 (18.7) | 0.147 |
| ***Change of airway operator*** |  |  |  |  | 0.285 |  |  |  | 0.328 |
| No change | 3305 (94.0) | 540 (93.1) | 128 (90.8) | 412 (93.8) |  | 2765 (94.1) | 784 (95.4) | 1981 (93.7) |  |
| 1 | 117 (3.3) | 20 (3.5) | 7 (5.0) | 13 (3.0) |  | 97 (3.3) | 21 (2.6) | 76 (3.6) |  |
| 2 | 2 (0.1) | 1 (0.2) | 1 (0.7) | 0 (0) |  | 1 (0.0) | 0 (0) | 1 (0.1) |  |
| >2 | 1 (0.0) | 1 (0.2) | 0 (0) | 1 (0.2) |  | 0 (0) | 0 (0) | 0 (0) |  |
| Missing | 92 (2.6) | 18 (3.1) | 5 (3.6) | 13 (3.0) |  | 74 (2.5) | 17 (2.1) | 57 (2.7) |  |

**Supplemental Table S3.** Additional characteristics of the airway operator

|  |  | **Emergency** | | | | **Non-Emergency** | | | |
| --- | --- | --- | --- | --- | --- | --- | --- | --- | --- |
|  | **All** | **All** | **DL** | **VL** | ***P*** | **All** | **DL** | **VL** | ***P*** |
|  | ***N=3517*** | ***N=580*** | ***N=141*** | ***N=439*** |  | ***N=2937*** | ***N=822*** | ***N=2115*** |  |
| ***Level of definitive airway operator*** | |  |  |  | 0.003 |  |  |  | 0.001 |
| Senior Consultant Anaesthetist | 160 (4.6) | 42 (7.2) | 13 (9.2) | 29 (6.6) |  | 118 (4.0) | 45 (5.5) | 73 (3.5) |  |
| Consultant Anaesthetist | 485 (13.8) | 115 (19.8) | 32 (22.7) | 83 (18.9) |  | 370 (12.6) | 103 (12.5) | 267 (12.6) |  |
| Anaesthesia resident | 1394 (39.6) | 202 (34.8) | 31 (22.0) | 171 (39.0) |  | 1192 (40.6) | 294 (35.8) | 898 (42.5) |  |
| Anaesthesia nurse | 1477 (42.0) | 221 (38.1) | 65 (46.1) | 156 (35.5) |  | 1256 (42.8) | 380 (46.2) | 876 (41.4) |  |
| ***Years of experience (consultants)*** | |  |  |  | 0.421 |  |  |  | 0.067 |
| 1-4 years | 312 (65.1) | 82 (72.6) | 21 (65.6) | 61 (75.3) |  | 230 (62.8) | 56 (54.9) | 174 (65.9) |  |
| >5 years | 167 (34.9) | 31 (27.4) | 11 (34.4) | 20 (24.7) |  | 136 (37.2) | 46 (45.1) | 90 (34.1) |  |
| ***Years of experience (resident)*** | |  |  |  | 0.979 |  |  |  | 0.008 |
| 1-2 years | 448 (32.2) | 55 (27.2) | 9 (29.0) | 46 (26.9) |  | 393 (33.1) | 78 (26.6) | 315 (35.2) |  |
| 3-5 years | 942 (67.8) | 147 (72.8) | 22 (71.0) | 125 (73.1) |  | 795 (66.9) | 215 (73.4) | 580 (64.8) |  |
| ***Education level anaesthesia nurse*** | |  |  |  | >0.99 |  |  |  | 0.531 |
| Certified | 1329 (90) | 216 (97.7) | 64 (98.5) | 152 (97.4) |  | 1113 (88.6) | 333 (87.6) | 780 (89.0) |  |
| In training | 148 (10) | 5 (2.3) | 1 (1.5) | 4 (2.6) |  | 143 (11.4) | 47 (12.4) | 96 (11.0) |  |

Data given in number (percent)

**Supplemental Table S4.** Area under the receiver operating characteristic (AUROC) for the primary outcomes, *first-attempt success*, and *difficulty of intubation* for the individual and cumulative HEAVEN criteria model for the emergency and non-emergency cohort undergoing rapid sequence intubation with direct (DL) or video laryngoscopy (VL)

| **Cohort** | **Device** | **First-attempt success** | **Difficulty of intubation** |
| --- | --- | --- | --- |
| *Cumulative HEAVEN criteria* |  |  |  |
| Overall cohort | All | 0.65 (95%-CI: 0.61 - 0.69) | 0.73 (95%-CI: 0.69 - 0.76) |
| Emergency cohort | All | 0.68 (95%-CI: 0.60 - 0.76) | 0.75 (95%-CI: 0.68 - 0.81) |
|  | DL | 0.70 (95%-CI: 0.58 - 0.83) | 0.66 (95%-CI: 0.54 - 0.79) |
|  | VL | 0.68 (95%-CI: 0.57 - 0.79) | 0.78 (95%-CI: 0.70 - 0.85) |
| Non-Emergency cohort | All | 0.64 (95%-CI: 0.60 - 0.69) | 0.72 (95%-CI: 0.68 - 0.75) |
|  | DL | 0.60 (95%-CI: 0.50 - 0.69) | 0.72 (95%-CI: 0.63 - 0.80) |
|  | VL | 0.66 (95%-CI: 0.61 - 0.71) | 0.71 (95%-CI: 0.67 - 0.76) |
| *Individual HEAVEN criteria* |  |  |  |
| Overall cohort | All | 0.69 (95%-CI: 0.64 - 0.73) | 0.77 (95%-CI: 0.73 - 0.80) |
| Emergency cohort | All | 0.77 (95%-CI: 0.69 - 0.85) | 0.80 (95%-CI: 0.74 - 0.86) |
|  | DL | 0.82 (95%-CI: 0.71 - 0.93) | 0.81 (95%-CI: 0.69 - 0.92) |
|  | VL | 0.77 (95%-CI: 0.67 - 0.86) | 0.82 (95%-CI: 0.74 - 0.89) |
| Non-Emergency cohort | All | 0.67 (95%-CI: 0.63 - 0.72) | 0.76 (95%-CI: 0.72 - 0.80) |
|  | DL | 0.65 (95%-CI: 0.55 - 0.75) | 0.79 (95%-CI: 0.70 - 0.88) |
|  | VL | 0.68 (95%-CI: 0.62 - 0.73) | 0.75 (95%-CI: 0.71 - 0.80) |

**Supplemental Table S5.** Sensitivity, specificity, positive prediction value (PPV), and negative prediction value (NPV) of the HEAVEN criteria for the primary outcomes, *first-attempt success*, and *difficulty of intubation* for the individual and cumulative HEAVEN criteria model for the emergency and non-emergency cohort undergoing rapid sequence intubation with direct or video laryngoscopy

| **Cohort** | **Device** | **Metric** | **First-attempt success** | **Difficulty of intubation** |
| --- | --- | --- | --- | --- |
| *Cumulative HEAVEN criteria* | | | | |
| Overall cohort | All | SENS | 100.0% (95%-CI: 99.9% - 100.0%) | 0.0% (95%-CI: 0.0% - 1.8%) |
|  |  | SPEC | 0.0% (95%-CI: 0.0% - 2.1%) | 100.0% (95%-CI: 99.9% - 100.0%) |
|  |  | PPV | 95.0% (95%-CI: 94.2% - 95.7%) | NaN% (95%-CI: 0.0% - 100.0%) |
|  |  | NPV | NaN% (95%-CI: 0.0% - 100.0%) | 94.2% (95%-CI: 93.4% - 95.0%) |
| Emergency cohort | All | SENS | 100.0% (95%-CI: 99.3% - 100.0%) | 0.0% (95%-CI: 0.0% - 6.6%) |
|  |  | SPEC | 0.0% (95%-CI: 0.0% - 9.7%) | 100.0% (95%-CI: 99.3% - 100.0%) |
|  |  | PPV | 93.8% (95%-CI: 91.5% - 95.6%) | NaN% (95%-CI: 0.0% - 100.0%) |
|  |  | NPV | NaN% (95%-CI: 0.0% - 100.0%) | 90.7% (95%-CI: 88.0% - 92.9%) |
|  | DL | SENS | 100.0% (95%-CI: 97.2% - 100.0%) | 0.0% (95%-CI: 0.0% - 24.7%) |
|  |  | SPEC | 0.0% (95%-CI: 0.0% - 28.5%) | 100.0% (95%-CI: 97.2% - 100.0%) |
|  |  | PPV | 92.2% (95%-CI: 86.5% - 96.0%) | NaN% (95%-CI: 0.0% - 100.0%) |
|  |  | NPV | NaN% (95%-CI: 0.0% - 100.0%) | 90.8% (95%-CI: 84.7% - 95.0%) |
|  | VL | SENS | 100.0% (95%-CI: 99.1% - 100.0%) | 0.0% (95%-CI: 0.0% - 8.6%) |
|  |  | SPEC | 0.0% (95%-CI: 0.0% - 13.7%) | 100.0% (95%-CI: 99.1% - 100.0%) |
|  |  | PPV | 94.3% (95%-CI: 91.7% - 96.3%) | NaN% (95%-CI: 0.0% - 100.0%) |
|  |  | NPV | NaN% (95%-CI: 0.0% - 100.0%) | 90.7% (95%-CI: 87.5% - 93.2%) |
| Non-Emergency cohort | All | SENS | 100.0% (95%-CI: 99.9% - 100.0%) | 0.0% (95%-CI: 0.0% - 2.4%) |
|  |  | SPEC | 0.0% (95%-CI: 0.0% - 2.6%) | 100.0% (95%-CI: 99.9% - 100.0%) |
|  |  | PPV | 95.2% (95%-CI: 94.4% - 96.0%) | NaN% (95%-CI: 0.0% - 100.0%) |
|  |  | NPV | NaN% (95%-CI: 0.0% - 100.0%) | 94.9% (95%-CI: 94.1% - 95.7%) |
|  | DL | SENS | 100.0% (95%-CI: 99.5% - 100.0%) | 0.0% (95%-CI: 0.0% - 12.8%) |
|  |  | SPEC | 0.0% (95%-CI: 0.0% - 11.6%) | 100.0% (95%-CI: 99.5% - 100.0%) |
|  |  | PPV | 96.4% (95%-CI: 94.8% - 97.5%) | NaN% (95%-CI: 0.0% - 100.0%) |
|  |  | NPV | NaN% (95%-CI: 0.0% - 100.0%) | 96.7% (95%-CI: 95.3% - 97.8%) |
|  | VL | SENS | 100.0% (95%-CI: 99.8% - 100.0%) | 0.0% (95%-CI: 0.0% - 3.0%) |
|  |  | SPEC | 0.0% (95%-CI: 0.0% - 3.3%) | 100.0% (95%-CI: 99.8% - 100.0%) |
|  |  | PPV | 94.8% (95%-CI: 93.8% - 95.7%) | NaN% (95%-CI: 0.0% - 100.0%) |
|  |  | NPV | NaN% (95%-CI: 0.0% - 100.0%) | 94.2% (95%-CI: 93.2% - 95.2%) |
| *Individual HEAVEN criteria* | | | | |
| Overall cohort | All | SENS | 100.0% (95%-CI: 99.8% - 100.0%) | 4.9% (95%-CI: 2.4% - 8.9%) |
|  |  | SPEC | 1.1% (95%-CI: 0.1% - 4.0%) | 99.8% (95%-CI: 99.6% - 99.9%) |
|  |  | PPV | 95.0% (95%-CI: 94.3% - 95.7%) | 58.8% (95%-CI: 32.9% - 81.6%) |
|  |  | NPV | 66.7% (95%-CI: 9.4% - 99.2%) | 94.5% (95%-CI: 93.7% - 95.2%) |
| Emergency cohort | All | SENS | 99.8% (95%-CI: 99.0% - 100.0%) | 11.1% (95%-CI: 4.2% - 22.6%) |
|  |  | SPEC | 11.1% (95%-CI: 3.1% - 26.1%) | 99.2% (95%-CI: 98.1% - 99.8%) |
|  |  | PPV | 94.4% (95%-CI: 92.2% - 96.2%) | 60.0% (95%-CI: 26.2% - 87.8%) |
|  |  | NPV | 80.0% (95%-CI: 28.4% - 99.5%) | 91.6% (95%-CI: 89.0% - 93.7%) |
|  | DL | SENS | 100.0% (95%-CI: 97.2% - 100.0%) | 15.4% (95%-CI: 1.9% - 45.4%) |
|  |  | SPEC | 9.1% (95%-CI: 0.2% - 41.3%) | 100.0% (95%-CI: 97.2% - 100.0%) |
|  |  | PPV | 92.9% (95%-CI: 87.3% - 96.5%) | 100.0% (95%-CI: 15.8% - 100.0%) |
|  |  | NPV | 100.0% (95%-CI: 2.5% - 100.0%) | 92.1% (95%-CI: 86.3% - 96.0%) |
|  | VL | SENS | 99.8% (95%-CI: 98.7% - 100.0%) | 12.2% (95%-CI: 4.1% - 26.2%) |
|  |  | SPEC | 16.0% (95%-CI: 4.5% - 36.1%) | 98.7% (95%-CI: 97.1% - 99.6%) |
|  |  | PPV | 95.2% (95%-CI: 92.7% - 97.0%) | 50.0% (95%-CI: 18.7% - 81.3%) |
|  |  | NPV | 80.0% (95%-CI: 28.4% - 99.5%) | 91.6% (95%-CI: 88.6% - 94.1%) |
| Non-Emergency | All | SENS | 100.0% (95%-CI: 99.8% - 100.0%) | 1.3% (95%-CI: 0.2% - 4.8%) |
|  |  | SPEC | 1.4% (95%-CI: 0.2% - 5.1%) | 100.0% (95%-CI: 99.8% - 100.0%) |
|  |  | PPV | 95.3% (95%-CI: 94.5% - 96.0%) | 66.7% (95%-CI: 9.4% - 99.2%) |
|  |  | NPV | 66.7% (95%-CI: 9.4% - 99.2%) | 95.0% (95%-CI: 94.1% - 95.8%) |
|  | DL | SENS | 100.0% (95%-CI: 99.5% - 100.0%) | 0.0% (95%-CI: 0.0% - 12.8%) |
|  |  | SPEC | 0.0% (95%-CI: 0.0% - 11.6%) | 100.0% (95%-CI: 99.5% - 100.0%) |
|  |  | PPV | 96.4% (95%-CI: 94.8% - 97.5%) | NaN% (95%-CI: 0.0% - 100.0%) |
|  |  | NPV | NaN% (95%-CI: 0.0% - 100.0%) | 96.7% (95%-CI: 95.3% - 97.8%) |
|  | VL | SENS | 100.0% (95%-CI: 99.7% - 100.0%) | 1.6% (95%-CI: 0.2% - 5.8%) |
|  |  | SPEC | 2.7% (95%-CI: 0.6% - 7.8%) | 99.9% (95%-CI: 99.7% - 100.0%) |
|  |  | PPV | 94.9% (95%-CI: 93.9% - 95.8%) | 66.7% (95%-CI: 9.4% - 99.2%) |
|  |  | NPV | 75.0% (95%-CI: 19.4% - 99.4%) | 94.3% (95%-CI: 93.2% - 95.3%) |

Abbreviations: NaN, Not a Number; Given the low incidence of patients presenting with >1 HEAVEN criteria, the calculation of PPV of *first-attempt success* and NPV of *difficulty during intubation* within the cumulative model was not possible.

**Supplemental Table S6.** Airway operator characteristics for paediatric and adult patients in the emergency and non-emergency cohorts

|  | **Emergency** | | | **Non-Emergency** | | |
| --- | --- | --- | --- | --- | --- | --- |
|  | **<16 yr** | ≥**16 yr** | ***P*** | **<16 yr** | ≥**16 yr** | ***P*** |
|  | ***N=29*** | ***N=551*** |  | ***N=303*** | ***N=2633*** |  |
| *Level of definitive airway operator* |  |  | 0.119 |  |  | 0.020 |
| Senior consultant anaesthetist | 5 (17.2) | 37 (6.7) |  | 22 (7.3) | 96 (3.7) |  |
| Consultant anaesthetist | 6 (20.7) | 109 (19.8) |  | 41 (13.5) | 329 (12.5) |  |
| Anaesthesia resident | 11 (37.9) | 191 (34.7) |  | 118 (38.9) | 1074 (40.8) |  |
| Anaesthesia Nurse | 7 (24.1) | 214 (38.8) |  | 122 (40.3) | 1134 (43.1) |  |
| *Years of experience (consultants)* |  |  | 0.047 |  |  | 0.032 |
| 1-4 years | 2 (33.3) | 80 (74.8) |  | 19 (46.3) | 211 (64.9) |  |
| >5 years | 4 (66.7) | 27 (25.2) |  | 22 (53.7) | 114 (35.1) |  |
| *Years of experience (resident)* |  |  | 0.731 |  |  | 0.001 |
| 1-2 years | 2 (18.2) | 53 (27.7) |  | 22 (18.6) | 371 (34.7) |  |
| 3-5 years | 9 (81.8) | 138 (72.3) |  | 96 (81.4) | 699 (65.3) |  |
| *Education level anaesthesia nurse* |  |  | 1.000 |  |  | 0.309 |
| Certified | 7 (100) | 209 (97.7) |  | 112 (91.8) | 1001 (88.3) |  |
| Trainee | 0 (0.0) | 5 (2.3) |  | 10 (8.2) | 133 (11.7) |  |

**Supplemental Figure S1.** Study Flow Chart

*Excluded:*

N=30 Patient in cardiac arrest

N=24 Missing number of attempts

N=441 Missing difficulty of intubation

All rapid sequence intubation (RSI)

N=4994

Eligible RSI

N=4003

*Excluded:*

N=991 Existing refusal of consent

Analysed RSI

N=3517


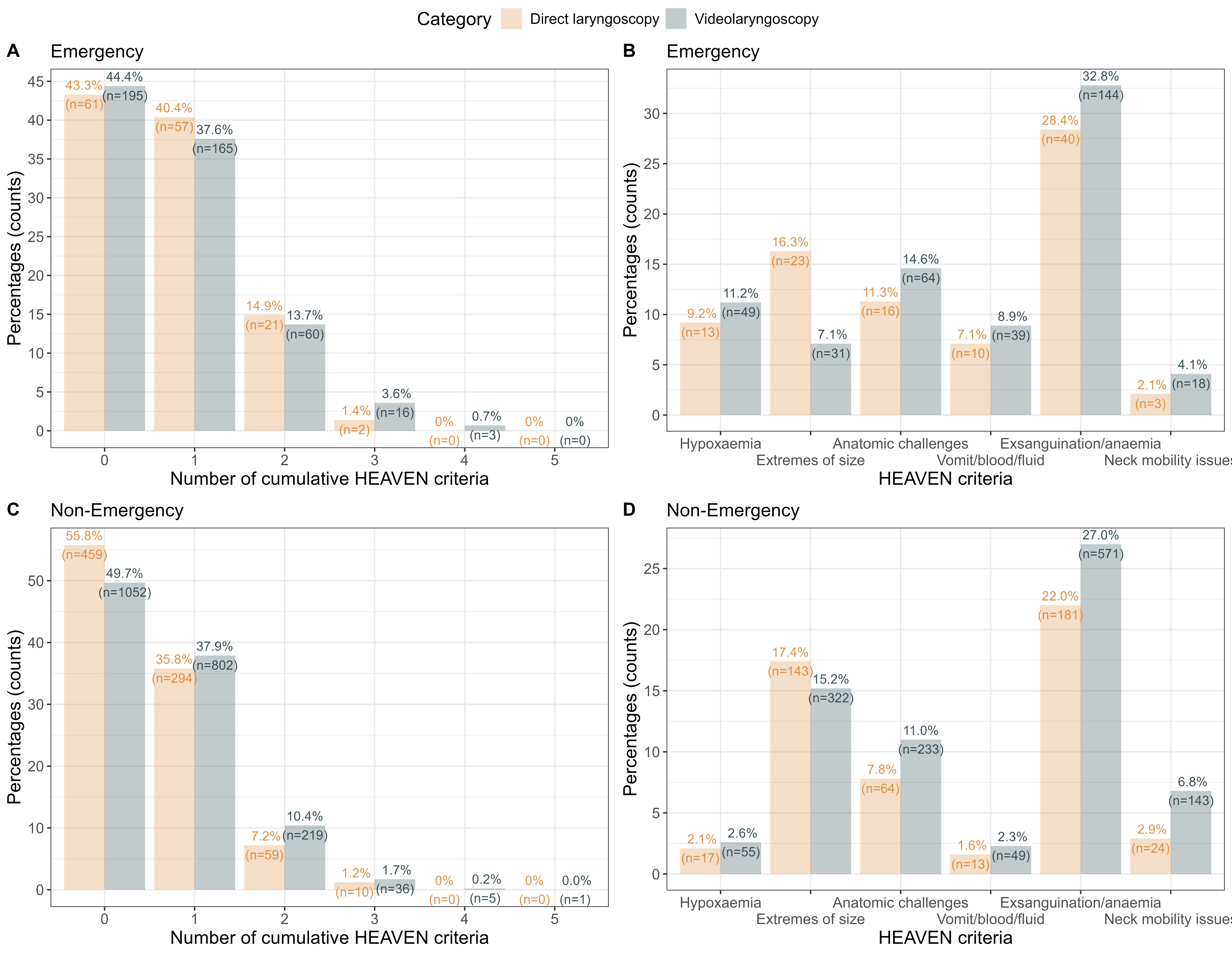


**Supplemental Figure S2.** The distribution of cumulative HEAVEN criteria (A, C) and the number and percentage of each criterion for emergency and non-emergency patients (B, D), both according to the intubation device (video or direct laryngoscope)


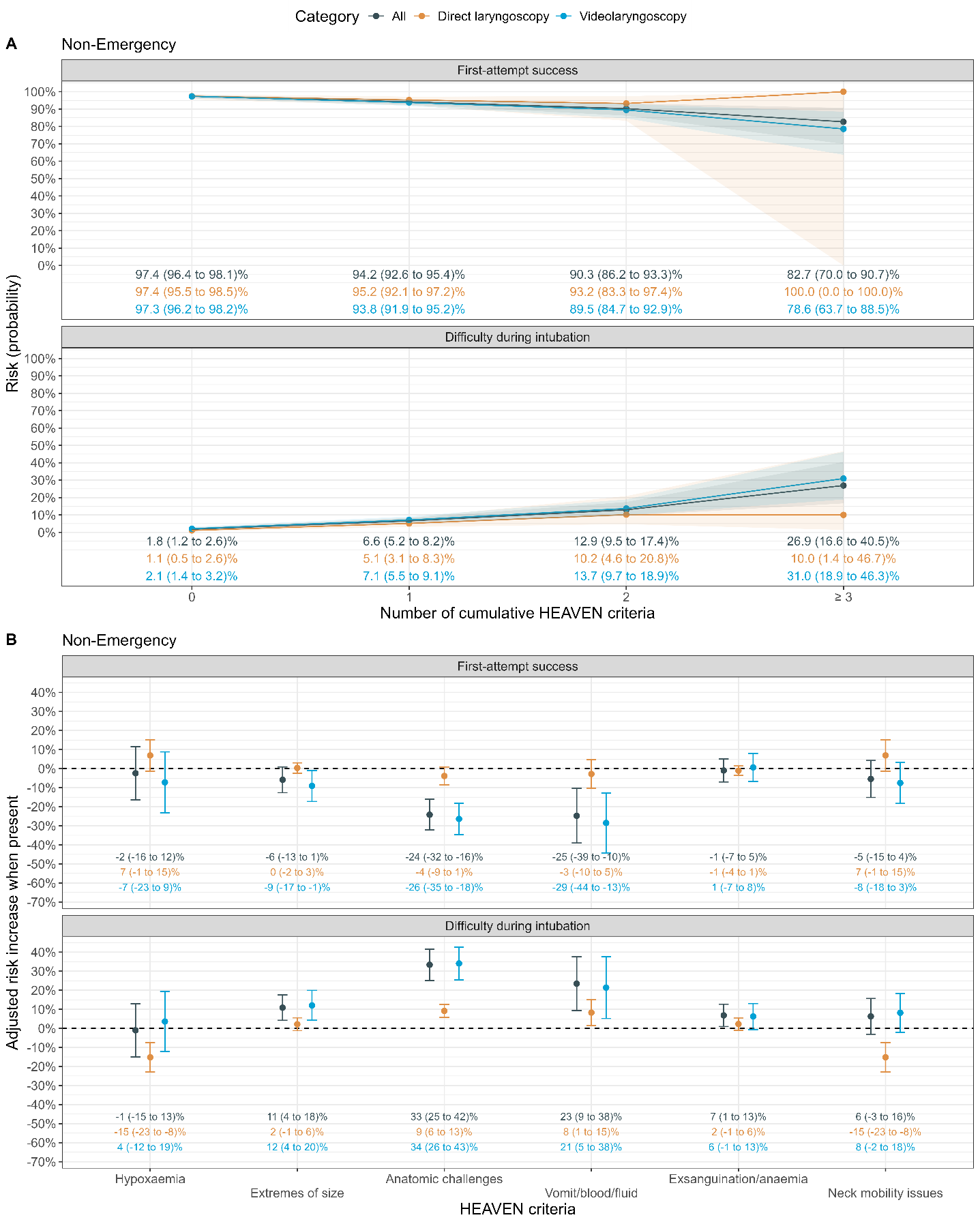
**Supplemental Figure S3.** **A**: Risk (probability) of first-attempt success and difficulties during rapid sequence intubation in emergency patients for the overall cohort and stratified by direct and video laryngoscopy across increasing numbers of cumulative HEAVEN criteria. Increasing numbers of HEAVEN criteria are associated with lower first pass success and higher risk of difficult airway. **B:** Adjusted risk increase associated with each individual HEAVEN criterion for first pass success and difficult airway in emergency patients, stratified by intubation device. **Panel A** is based on a prediction model including the cumulative number of HEAVEN criteria (cumulative model), and **Panel B** on a model including individual HEAVEN criteria (individual model). Data are presented as mean risk estimates with 95% confidence intervals; corresponding numerical values are shown below each panel. Wide 95% confidence intervals for direct laryngoscopy with more than three criteria indicate infrequent use of this device in such cases and limit interpretation.

**
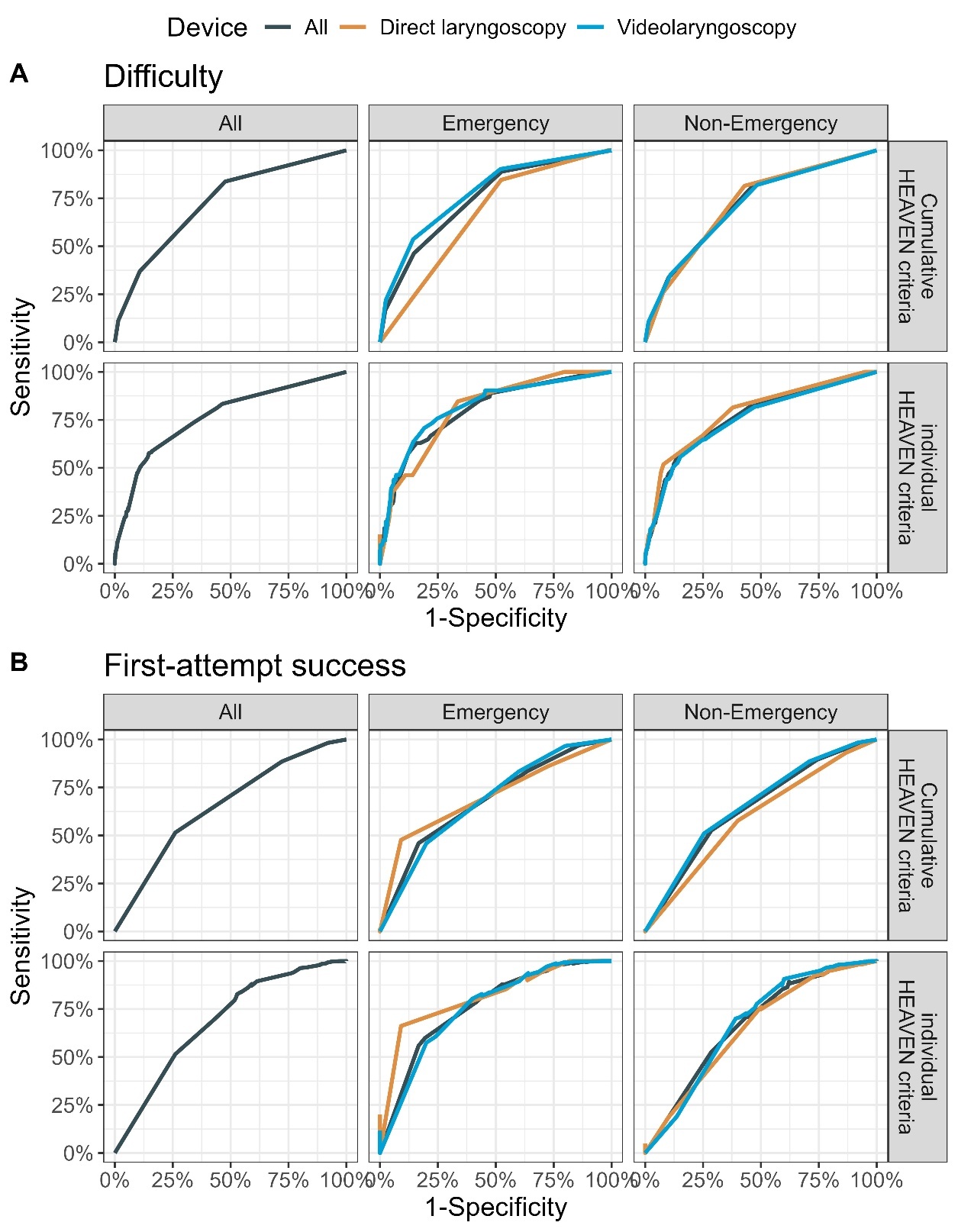
**

**Supplemental Figure S4.** Area under the receiver operating characteristic (AUROC) for the primary outcomes (A) *difficulty of intubation*, and (B) *first-attempt success* for the individual and cumulative HEAVEN criteria model.
